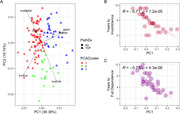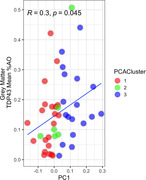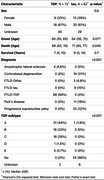# Pathological Associations with Rate of Progression of Frontotemporal Dementia

**DOI:** 10.1002/alz.089185

**Published:** 2025-01-03

**Authors:** Rogan Magee, Daniel T Ohm, Emma Rhodes, Lauren Massimo, David A Wolk, Eddie B Lee, Corey T McMillan, David J Irwin

**Affiliations:** ^1^ Department of Neurology, Perelman School of Medicine, University of Pennsylvania, Philadelphia, PA USA; ^2^ Frontotemporal Degeneration Center, Department of Neurology, Perelman School of Medicine, University of Pennsylvania, Philadelphia, PA USA; ^3^ Frontotemporal Degeneration Center, University of Pennsylvania, Philadelphia, PA USA; ^4^ Penn Frontotemporal Degeneration Center, Department of Neurology, Perelman School of Medicine, University of Pennsylvania, Philadelphia, PA USA; ^5^ Department of Neurology, University of Pennsylvania, Philadelphia, PA USA; ^6^ Center for Neurodegenerative Disease Research, University of Pennsylvania, Philadelphia, PA USA

## Abstract

**Background:**

There is considerable variability in the rate of clinical progression among individuals with frontotemporal dementia (FTD) and prognostic markers are lacking. Moreover, due to the rarity of postmortem data, the relationship between rate of progression and postmortem tau and TDP‐43 proteinopathy is understudied.

**Method:**

To explore the pathologic underpinnings of differences in clinical progression of FTD, we used clinical data collected by the Penn Center for Neurodegenerative Disease Research from 130 patients with autopsy‐confirmed frontotemporal lobar degeneration (FTLD‐tau = 62, FTLD‐TDP = 68) across six domains (age at onset, survival in years, first Clinical Dementia Rating [CDR] scale score, first Mini‐Mental State Examination [MMSE] score, annual change in CDR, annual change in MMSE). We used principal components analysis to scale and collapse these data into two dimensions, and k means to automatically select three clusters based on silhouette width. We used automated digital pathology to measure mean percent area occupied (AO%) by TDP‐43 pathologic inclusions in up to 17 distinct autopsied brain regions.

**Result:**

PCA (Fig 1A) Cluster 3 progressed relatively fastest and was enriched for FTLD‐TDP cases and GRN mutation carriers (Table 1). Among FTLD‐TDP cases, TDP type E was enriched in cluster 3 (3 of 4) and type C was enriched in cluster 1 (13 of 16). Among FTLD‐tau cases, progressive supranuclear palsy was overrepresented in cluster 1 (19 of 20). Principal component 1 (PC1) negatively correlated with independent measures of functional outcomes extracted from the clinical record, including the time to develop incontinence and complete dependence in activities of daily living (Figure 1B, C). PC1 correlated with the average pathologic TDP‐43 AO% in FTLD‐TDP cases (Figure 2).

**Conclusion:**

Taken together, these results suggest that increased rate of clinical progression may relate to increased burden of TDP‐43 observed postmortem. The heterogeneity in the prognosis of FTLD may be driven by biological factors, such as disease activity. Further, FTLD‐TDP (especially FTLD‐GRN) may associate with faster progression. Biomarkers of FTLD‐TDP and/or improved access to mutation testing may improve prognostication in the care of FTD.